# Distinct Associations of Hedonic and Eudaimonic Motives with Well-Being: Mediating Role of Self-Control

**DOI:** 10.3390/ijerph17155547

**Published:** 2020-07-31

**Authors:** Zhijia Zeng, Hezhi Chen

**Affiliations:** 1 Student Affairs Department, Zhejiang University of Finance and Economics, Hangzhou 310018, China; 2 Department of Psychology and Behavioral Sciences, Zhejiang University, Hangzhou 310058, China

**Keywords:** hedonic motives, eudaimonic motives, self-control, well-being, happiness

## Abstract

The pursuit of hedonia and eudaimonia are two ways to fulfill the goal of a “good life”. While some studies report that both hedonic and eudaimonic motives improve well-being, others suggest that hedonic motives are counterproductive, raising the question of whether and why eudaimonic motives are more positively associated with well-being. We aimed to identify the distinct associations of hedonic and eudaimonic motives with well-being and investigate whether they are partly mediated by self-control. A total of 2882 college freshmen (1835 females, 1047 males, mean age 18.16 years) completed measures assessing hedonic and eudaimonic motives, self-control, life satisfaction, positive and negative affect, and eudaimonic well-being. Eudaimonic motives were associated with higher life satisfaction, more positive affect, less negative affect, and better eudaimonic well-being. In contrast, hedonic motives were positively associated with life satisfaction, while also being correlated with a greater degree of negative affect and impaired eudaimonic well-being. Self-control mediated the relationships between hedonic and eudaimonic motives and well-being. Eudaimonic and hedonic motives were positively and negatively related to self-control, respectively. Further, high self-control was associated with greater life satisfaction, positive affect, and eudaimonic well-being and lower negative affect. Thus, eudaimonic motives can lead to a better life than hedonic motives because the former enhance self-control, while the latter lower it.

## 1. Introduction

The pursuit of a good life is a fundamental motive for human beings. However, individuals can have very different ideas about what constitutes a “good life”. Specifically, the two most prominent views of a good life are the hedonic view and the eudaimonic view [1]. Pursuing hedonia, or hedonic motives, involves seeking personal enjoyment, pleasure, and comfort; pursuing eudaimonia, or eudaimonic motives, relates to seeking personal growth, excellence, meaning, and authenticity [2].

### 1.1. Hedonic and Eudaimonic Motives and Well-Being

One issue that has received much scholarly attention is how hedonic and eudaimonic motives affect well-being. Aristotle claimed that people should strive for a virtuous life rather than seeking pleasure, because the latter does not bring happiness. Consistent with this view, numerous studies have suggested that while eudaimonic motives generally improve well-being, pursuing hedonia does not result in the achievement of the goal of a “good life” and might even backfire [3,4,5]. For example, in one study, eudaimonic motives were correlated with various well-being outcomes, including life satisfaction, vitality, positive affect, negative affect, carefreeness, self-connectedness, and meaning, whereas hedonic motives only predicted vitality, positive affect, and carefreeness [4]. In another study, eudaimonic motives were negatively correlated with depression and stress; in contrast, there was no significant correlation between hedonic motives and depression or stress [5]. Sheldon et al. [3] further showed that participants’ motivations for improving their subjective well-being were negatively correlated with concurrent subjective well-being and did not affect longitudinal subjective well-being.

However, some studies have reported the well-being-related benefits of both hedonic and eudaimonic motives [6,7,8]. For example, Peterson et al. [8] showed that endorsement of pleasure, engagement, and meaning as paths to happiness all predicted life satisfaction. Similarly, Huta and Ryan [6] showed that hedonic and eudaimonic motives had both overlapping and distinct effects on well-being; hedonic motives related more to positive affect and life satisfaction, while eudaimonic motives related more to meaning. Overall, a combination of hedonic and eudaimonic motives was associated with the greatest well-being.

In sum, whether hedonic and eudaimonic motives lead to different well-being outcomes remains inadequately explored. Further, the factors that account for the potential distinct associations of hedonic and eudaimonic motives with well-being remain unclear.

### 1.2. Hedonic and Eudaimonic Motives and Self-Control

We argue that one factor that could account for the distinct associations of hedonic and eudaimonic motives with well-being is self-control. Self-control is defined as the ability to override one’s own inner responses or interrupt a course of action, especially when conflicting with ideals, values, and social expectations, and to support the fulfillment of long-term goals [9,10]. Huta [11] pointed out two crucial distinctions between hedonic and eudaimonic motives. First, hedonic motives are concerned with satiating one’s own needs and desires in the present or near future; in contrast, eudaimonic motives are concerned with fulfilling long-term objectives. The second difference is that hedonic motives are concerned with what feels good, whereas eudaimonic motives relate to what one believes to be right. According to the self-concordance model, individuals will strive for goals more persistently when they fit their core values [12,13]. Similarly, the goal theory of happiness suggests that individuals actively engage in behaviors that are in line with their identified goals, which, in turn, further influence their well-being [14,15]. Consequently, when faced with conflicts such as those between short-term desires and long-term objectives, individuals pursuing eudaimonia are more likely to implement a higher level of self-control to achieve their goals than are those pursuing hedonia.

Although empirical research on the relationships of hedonic and eudaimonic motives with self-control is sparse, two studies have provided some supporting evidence [7,16]. Peterson et al. [16] showed that endorsement of engagement and meaning as routes to happiness was positively related to self-regulation. More recently, Anic and Tončić [7] showed that individuals pursuing eudaimonia but not hedonia had a high level of self-control, whereas those pursuing hedonia but not eudaimonia had a relatively low level of self-control.

### 1.3. Self-Control and Well-Being

Studies have indicated that self-control positively predicts hedonic well-being [17,18,19,20,21]. For example, Briki [18] showed that self-control was positively related to happiness and life satisfaction. Hofmann et al. [17] found that self-control promoted affective experience and life satisfaction by managing goal conflict and facilitating the achievement of multiple goals. Similarly, Cheung et al. [19] demonstrated that people with higher self-control were happier because they were more promotion-focused and less prevention-focused. In addition, a lack of self-control has been linked to various problematic behaviors, such as procrastination, impulsive purchasing, addictive behavior, and even criminal offenses [22,23,24,25], which, in turn, might increase depression, stress, guilt, and other negative feelings [15,26,27].

Furthermore, it has been suggested that one approach to enhance eudaimonic well-being is satisfying basic psychological needs, including autonomy, competence, and relatedness [28]. Self-control itself is essential to the attainment of autonomy [29]. In addition, self-control has been found to be associated with higher achievement and better interpersonal relationships [20,30,31], which might satisfy the need for competence and relatedness. Consequently, self-control might also result in greater eudaimonic well-being.

### 1.4. The Current Study

The aim of this study is to identify the distinct associations between hedonic and eudaimonic motives and well-being outcomes, and further investigate the possible mediating role of self-control. For this purpose, we collected cross-sectional data on hedonic and eudaimonic motives, self-control, hedonic well-being (life satisfaction, positive and negative affect), and eudaimonic well-being. We propose the following hypotheses:

**Hypothesis** **1.** *Self-control mediates the relationships between hedonic motives and well-being outcomes. Hedonic motives are detrimental to well-being outcomes by lowering the self-control of Chinese college students.*

**Hypothesis** **2.** *Self-control mediates the relationships between eudaimonic motives and well-being outcomes. Eudaimonic motives promote well-being outcomes by increasing the self-control of Chinese college students.*

## 2. Materials and Methods

### 2.1. Sample and Procedures

In September 2019, we carried out a cross-sectional survey with a sample of 2882 students (mean age = 18.16, standard deviation = 0.44; 1047 males, 1835 females) from Zhejiang University of Finance and Economics. The participants were freshmen who had to complete a general psychological survey for course credits. The students completed the online survey while seated in individual cubicles. The teachers gave instructions and maintained discipline. All responses were included in the analyses.

The study design and data collection procedures were both approved by the Ethics Committee of Zhejiang University of Finance and Economics. All students provided written informed consent.

### 2.2. Measures

All the scales used in the current study were originally in English. Therefore, all the survey measures were translated from English to Chinese and back to English. When there were discrepancies between the original English version and the back-translated English version, those items were further modified after discussion by the two authors.

#### 2.2.1. Hedonic and Eudaimonic Motives

To assess motives, we used the Hedonic and Eudaimonic Motives for Activities—Revised scale developed by Huta [32]. This instrument utilizes five items to assess hedonic motives (e.g., “seeking pleasure”) and five for eudaimonic motives (e.g., “seeking to use the best in yourself”). Respondents were asked to report the degrees of each motive with which they typically approached their activities on a seven-point Likert scale ranging from 1 (not at all) to 7 (very much). The Cronbach’s α values were 0.83 for hedonic motives and 0.79 for eudaimonic motives.

#### 2.2.2. Self-Control

To assess personal self-control, we used the 13-item Brief Self-Control Scale [20]. A sample item is “I am good at resisting temptation.” Respondents indicated the degree to which each of the statements reflected their typical behavior on a five-point Likert scale ranging from 1 (not at all) to 5 (very much). The Cronbach’s α value was 0.82.

#### 2.2.3. Well-Being Outcomes

To assess life satisfaction, we used the Satisfaction with Life Scale, which has five items (e.g., “In most ways my life is close to my ideal”) [33] rated on a seven-point Likert scale ranging from 1 (strongly disagree) to 7 (strongly agree). The Cronbach’s α value was 0.84.

Participants also reported their positive and negative affect in the past two weeks on the Positive and Negative Affect Schedule on a five-point Likert scale ranging from 1 (not at all) to 5 (very much) [34]. The Cronbach’s α values were 0.90 for both positive and negative affect.

Thereafter, they completed the Questionnaire for Eudaimonic Well-Being, which comprises 21 items assessing various aspects of eudaimonic well-being, including self-discovery, perceived development of one’s potential, meaning in life, and involvement and enjoyment in personally expressive activities (e.g., “I find I get intensely involved in many of the things I do each day”) [35]. Participants indicated their agreement with each item on a five-point Likert scale ranging from 1 (strongly disagree) to 5 (strongly agree). The Cronbach’s α value was 0.81.

#### 2.2.4. Demographic Information

Demographic information included gender and age.

### 2.3. Analyses

All tests were performed using SPSS 23.0 software (IBM Corp., Armonk, NY, USA). First, descriptive statistics were used to describe hedonic and eudaimonic motives, self-control, and well-being outcomes. Next, we conducted correlational analyses to investigate the general relationships between hedonic and eudaimonic motives, self-control, and well-being outcomes. Furthermore, we tested the hypothesized mediation effects of hedonic and eudaimonic motives on well-being outcomes via self-control using the PROCESS macro for SPSS (model 4) [36,37]. Hedonic and eudaimonic motives were entered into the models as independent variables, self-control was entered as the mediating variable, and gender was entered as the control variable. The four well-being outcome variables were separately entered as dependent variables; thus, the mediation model was run four times. We reported hierarchical regression results, indirect effects, and the 95% bias-corrected confidence intervals (CIs) estimated by 5000 bootstrap samples. The indirect effect was considered significant if the 95% CI did not contain zero.

## 3. Results

### 3.1. Descriptive Analyses

Descriptive statistics related to hedonic and eudaimonic motives, self-control, and well-being outcomes are presented in Table 1. Bivariate correlations between the study variables are presented in Table 2. Higher hedonic motives were related to higher life satisfaction (*r* = 0.05, *p* = 0.004), but more negative affect (*r* = 0.15, *p* < 0.001) and worse eudaimonic well-being (*r* = −0.08, *p* < 0.001); there was no significant correlation between hedonic motives and positive affect (*r* = −0.01, *p* = 0.503). Higher eudaimonic motives were related to higher life satisfaction (*r* = 0.16, *p* < 0.001), more positive affect (*r* = 0.38, *p* < 0.001), less negative affect (*r* = −0.08, *p* = 0.001), and better eudaimonic well-being (*r* = 0.51, *p* < 0.001). Self-control was negatively related to hedonic motives (*r* = −0.25, *p* < 0.001) and positively related to eudaimonic motives (*r* = 0.26, *p* < 0.001). In addition, self-control was associated with all well-being outcomes (life satisfaction: *r* = 0.35, *p* < 0.001; positive affect: *r* = 0.38, *p* < 0.001; negative affect: *r* = −0.38, *p* < 0.001; and eudaimonic well-being: *r* = 0.50, *p* < 0.001).

### 3.2. Mediation Analyses

The regression analyses are shown in Table 3. As expected, after adjusting for gender, hedonic motives were negatively correlated with self-control (*ß* = −0.29, *p* < 0.001), while eudaimonic motives were positively correlated with self-control (*ß* = 0.30, *p* < 0.001). In addition, after controlling for hedonic and eudaimonic motives, self-control demonstrated significant positive associations with life satisfaction (*ß* = 0.37, *p* < 0.001), positive affect (*ß* = 0.30, *p* < 0.001), and eudaimonic well-being (*ß* = 0.37, *p* < 0.001) and a significant negative association with negative affect (*ß* = −0.37, *p* < 0.001). Whether gender was included as a control variable or not, the main results did not change.

The estimated direct and indirect effects between hedonic and eudaimonic motives and the four well-being outcomes are presented in Table 4. The indirect effects of hedonic motives on life satisfaction (indirect effect = −0.106, CI: (−0.123, −0.089)), positive affect (indirect effect = −0.054, CI: (−0.064, −0.045)), and eudaimonic well-being (indirect effect = −0.039, CI: (−0.045, −0.033)) via self-control were all negative, and the indirect effect of hedonic motives on negative affect (indirect effect = 0.067, CI: (0.056, 0.078)) via self-control was positive. In contrast, the indirect effects of eudaimonic motives on life satisfaction (indirect effect = 0.134, CI: (0.113, 0.156)), positive affect (indirect effect = 0.068, CI: (0.057, 0.081)), and eudaimonic well-being (indirect effect = 0.049, CI: (0.043, 0.056)) via self-control were all positive, and the indirect effect of eudaimonic motives on negative affect (indirect effect = −0.084, CI: (−0.098, −0.072)) via self-control was negative. This is consistent with the argument that hedonic motives could be detrimental to well-being outcomes by decreasing self-control, whereas eudaimonic motives promote well-being outcomes by increasing self-control.

## 4. Discussion

### 4.1. Findings and Implications

In this study, we examined the associations of hedonic and eudaimonic motives with well-being among Chinese college students. The current data demonstrated that, overall, eudaimonic motives were more positively associated with well-being than hedonic motives, which is consistent with previous studies [3]. Specifically, eudaimonic motives were found to be associated with improvements in all well-being outcomes, including higher life satisfaction, more positive affect, less negative affect, and better eudaimonic well-being. In contrast, the correlations of hedonic motives were more complex; while they were positively related to life satisfaction, they were not associated with positive affect and were even associated with an increase in negative affect. In addition, the pursuit of hedonia seemed to be detrimental to eudaimonic well-being.

More importantly, we investigated the mediating role of self-control, which shed light on the distinct associations of hedonic and eudaimonic motives with well-being. The results revealed that hedonic motives were detrimental to self-control, whereas eudaimonic motives could potentially enhance self-control, which is in accordance with previous results [7]. In addition, higher self-control was related to higher life satisfaction, more positive affect, less negative affect, and better eudaimonic well-being. In other words, eudaimonic motives generally promote well-being by enhancing self-control, whereas hedonic motives might be harmful by lowering self-control.

These findings highlight the potential detrimental effects of hedonic motives on well-being, which have been largely neglected in previous studies. Recent studies have shown that hedonic motives include both enjoyment and comfort [38,39,40], but only enjoyment motives might promote well-being [38]. Similarly, Huta [11] has suggested that while healthful approaches to hedonia would bring pleasure and enjoyment, excessive or unbalanced hedonic motives can have undesirable consequences. Combining previous findings with the current results, it can be suggested that the effects of hedonic motives on well-being are twofold. On the one hand, hedonic motives might encourage people to engage in entertaining activities, which can lead to improved well-being. On the other hand, hedonic motives could make people yield to temptation more easily, which might lead to dysfunctional behaviors such as addiction and procrastination, and further worsen well-being. Consequently, it is worthwhile to further explore functional and dysfunctional hedonic motives and hedonic behaviors.

### 4.2. Limitations and Future Research Directions

The present study has a number of limitations. First, we failed to implement an attention check, due to some accidental technical issues. Nevertheless, all scales achieved sufficient reliabilities. The distinct associations of hedonic and eudaimonic motives with well-being were unlikely to appear by chance. Consequently, we believe the results of the current study were reliable.

Second, the current data are correlational rather than causal. Future research should consider using longitudinal data to further determine the causal relationships between hedonic and eudaimonic motives, self-control, and well-being.

Third, the Questionnaire for Eudaimonic Well-Being is primarily a measure of functioning, whereas the other outcomes are all measures of experience. Further studies should use more suitable measures that represent eudaimonic experiences (e.g., a feeling of meaning) to replicate our findings.

Fourth, this paper only examines the mediating role of self-control in the relationships between hedonic and eudaimonic motives and well-being outcomes. However, as mentioned above, hedonic motives could encourage both healthful hedonic activities and dysfunctional behaviors. Investigating these factors simultaneously might facilitate a more comprehensive understanding of the effects of hedonic motives on well-being.

Last, our sample comprised solely Chinese college students. Prior research has suggested that cultural factors might moderate the relationships between hedonic and eudaimonic motives and well-being [41]. A future study direction would be exploring how and why the pursuit of happiness, especially hedonia, might affect well-being differently across cultures.

## 5. Conclusions

Despite the limitations mentioned above, the present study contributes to an improved understanding of the associations of hedonic and eudaimonic motives with well-being outcomes. In sum, hedonic motives could be detrimental to well-being by harming self-control; in contrast, eudaimonic motives can contribute to better well-being by improving self-control. These results suggest that the pursuit of eudaimonia is more likely to further the goal of leading a good life than the pursuit of hedonia.

## Figures and Tables

**Table 1:** Descriptive statistics for study variables.

Variables	Minimum	Maximum	Mean	SD
Hedonic motives	1.00	7.00	4.63	1.11
Eudaimonic motives	1.40	7.00	5.62	0.91
Self-control	1.15	4.92	2.98	0.59
Life satisfaction	1.00	7.00	4.17	1.09
Positive affect	1.00	5.00	3.35	0.69
Negative affect	1.00	5.00	2.10	0.69
Eudaimonic well-being	2.24	5.00	3.65	0.40

**Table 2:** Bivariate correlations between study variables.

TitleStudy Variables	1	2	3	4	5	6	7
1. Hedonic motives	-						
2. Eudaimonic motives	0.15 ***	-					
3. Self-control	−0.25 ***	0.26 ***	-				
4. Life satisfaction	0.05 **	0.16 ***	0.35 ***	-			
5. Positive affect	−0.01	0.38 ***	0.38 ***	0.46 ***	-		
6. Negative affect	0.15 ***	−0.08 **	−0.38 ***	−0.19 ***	−0.05 **	-	
7. Eudaimonic well-being	−0.08 ***	0.51 ***	0.50 ***	0.40 ***	0.53 ***	−0.32 ***	-

Note: ** *p* < 0.01, *** *p* < 0.001.

**Table 3:** Regression results.

Models	*B*	*ß*	*p*	*R* ^2^
**Dependent variable: Self-control**				0.15
Gender (female = 0, male = 1)	0.04	0.03	0.090	
Hedonic motives	−0.16	−0.29	<0.001	
Eudaimonic motives	0.20	0.30	<0.001	
**Depedent variable: Life satisfaction**				0.15
Gender (female = 0, male = 1)	0.13	0.06	0.001	
Hedonic motives	0.13	0.13	<0.001	
Eudaimonic motives	0.06	0.05	0.009	
Self-control	0.68	0.37	<0.001	
**Dependent variable: Positive affect**				0.24
Gender (female = 0, male = 1)	0.20	0.14	<0.001	
Hedonic motives	0.00	0.00	0.824	
Eudaimonic motives	0.23	0.30	<0.001	
Self-control	0.35	0.30	<0.001	
**Dependent variable: Negative affect**				0.15
Gender (female = 0, male = 1)	0.02	0.02	0.379	
Hedonic motives	0.03	0.06	0.003	
Eudaimonic motives	0.00	0.00	0.931	
Self-control	−0.43	−0.37	<0.001	
**Dependent variable: Eudaimonic well-being**				0.41
Gender (female = 0, male = 1)	0.04	0.05	0.002	
Hedonic motives	−0.02	−0.06	<0.001	
Eudaimonic motives	0.19	0.43	<0.001	
Self-control	0.25	0.37	<0.001	

**Table 4:** Direct and indirect effects and 95% confidence intervals.

Outcome Variables	Hedonic Motives		Eudaimonic Motives	
	Direct Effects	Indirect Effects	Direct Effects	Indirect Effects
Life satisfaction	0.130 (0.095, 0.165)	−0.106 (−0.123, −0.089)	0.057 (0.014, 0.101)	0.134 (0.113, 0.156)
Positive affect	0.002 (−0.019, 0.023)	−0.054 (−0.064, −0.045)	0.226 [0.200, 0.252)	0.068 [0.057, 0.081)
Negative affect	0.034 (0.012, 0.057)	0.067 (0.056, 0.078)	0.001 [−0.026, 0.029)	−0.084 [−0.098, −0.072)
Eudaimonic well-being	−0.022 (−0.032, −0.011)	−0.039 (−0.045, −0.033)	0.187 [0.174, 0.200)	0.049 [0.043, 0.056)

## References

[1] Ryan R.M., Deci E.L. (2001). On happiness and human potentials: A review of research on hedonic and eudaimonic well-being. Annu. Rev. Psychol..

[2] Huta V., Waterman A. (2014). Eudaimonia and its distinction from hedonia: Developing a classification and terminology for understanding conceptual and operational definitions. J. Happiness Stud..

[3] Sheldon K.M., Corcoran M., Prentice M. (2019). Pursuing eudaimonic functioning versus pursuing hedonic well-being: The first goal succeeds in its aim, whereas the second does not. J. Happiness Stud..

[4] Ortner C.N., Corno D., Fung T.Y., Rapinda K. (2018). The roles of hedonic and eudaimonic motives in emotion regulation. Personal. Individ. Differ..

[5] Kryza-Lacombe M., Tanzini E., O’Neill S. (2019). Hedonic and eudaimonic motives: Associations with academic achievement and negative emotional states among urban college students. J. Happiness Stud..

[6] Huta V., Ryan R.M. (2010). Pursuing pleasure or virtue: The differential and overlapping well-being benefits of hedonic and eudaimonic motives. J. Happiness Stud..

[7] Anic P., Tončić M. (2013). Orientations to happiness, subjective well-being and life goals. Psihol. Teme.

[8] Peterson C., Park N., Seligman M.E. (2005). Orientations to happiness and life satisfaction: The full life versus the empty life. J. Happiness Stud..

[9] Baumeister R.F., Vohs K.D., Tice D.M. (2007). The strength model of self-control. Curr. Dir. Psychol. Sci..

[10] Muraven M., Baumeister R.F. (2000). Self-regulation and depletion of limited resources: Does self-control resemble a muscle?. Psychol. Bull..

[11] Huta V. (2015). The complementary roles of eudaimonia and hedonia and how they can be pursued in practice. Posit. Psychol. Pract. Promot. Hum. Flourishing Work Health Educ. Everyday Life.

[12] Sheldon K. (2014). Becoming oneself: The central role of self-concordant goal selection. Personal. Soc. Psychol. Rev..

[13] Sheldon K.M., Elliot A.J. (1999). Goal striving, need satisfaction, and longitudinal well-being: The self-concordance model. J. Personal. Soc. Psychol..

[14] Fowers B.J., Mollica C.O., Procacci E.N. (2010). Constitutive and instrumental goal orientations and their relations with eudaimonic and hedonic well-being. J. Posit. Psychol..

[15] Yang Y., Li P., Fu X., Kou Y. (2017). Orientations to happiness and subjective well-being in Chinese adolescents: The roles of prosocial behavior and internet addictive behavior. J. Happiness Stud..

[16] Peterson C., Ruch W., Beermann U., Park N., Seligman M.E. (2007). Strengths of character, orientations to happiness, and life satisfaction. J. Posit. Psychol..

[17] Hofmann W., Luhmann M., Fisher R.R., Vohs K.D., Baumeister R.F. (2014). Yes, but are they happy? Effects of trait self-control on affective well-being and life satisfaction. J. Personal..

[18] Briki W. (2018). Trait self-control: Why people with a higher approach (avoidance) temperament can experience higher (lower) subjective wellbeing. Personal. Individ. Differ..

[19] Cheung T.T., Gillebaart M., Kroese F., De Ridder D. (2014). Why are people with high self-control happier? The effect of trait self-control on happiness as mediated by regulatory focus. Front. Psychol..

[20] Tangney J.P., Baumeister R.F., Boone A.L. (2004). High self-control predicts good adjustment, less pathology, better grades, and interpersonal success. J. Personal..

[21] Finkenauer C., Engels R., Baumeister R. (2005). Parenting behaviour and adolescent behavioural and emotional problems: The role of self-control. Int. J. Behav. Dev..

[22] Steel P. (2007). The nature of procrastination: A meta-analytic and theoretical review of quintessential self-regulatory failure. Psychol. Bull..

[23] Moffitt T.E., Arseneault L., Belsky D., Dickson N., Hancox R.J., Harrington H., Houts R., Poulton R., Roberts B.W., Ross S. (2011). A gradient of childhood self-control predicts health, weal th, and public safety. Proc. Natl. Acad. Sci. USA.

[24] Gottfredson M.R., Hirschi T. (1990). A General Theory of Crime.

[25] Baumeister R.F. (2002). Yielding to temptation: Self-control failure, impulsive purchasing, and consumer behavior. J. Consum. Res..

[26] Tice D.M., Baumeister R.F. (1997). Longitudinal study of procrastination, performance, stress, and health: The costs and benefits of dawdling. Psychol. Sci..

[27] Havassy B.E., Arns P.G. (1998). Relationship of cocaine and other substance dependence to well-being of high-risk psychiatric patients. Psychiatr. Serv..

[28] Deci E.L., Ryan R.M. (2008). Hedonia, eudaimonia, and well-being: An introduction. J. Happiness Stud..

[29] Ryan R.M., Huta V., Deci E.L. (2008). Living well: A self-determination theory perspective on eudaimonia. J. Happiness Stud..

[30] Duckworth A.L., Tsukayama E., May H. (2010). Establishing causality using longitudinal hierarchical linear modeling: An illustration predicting achievement from self-control. Soc. Psychol. Personal. Sci..

[31] Righetti F., Finkenauer C. (2011). If you are able to control yourself, I will trust you: The role of perceived self-control in interpersonal trust. J. Personal. Soc. Psychol..

[32] Huta V. (2016). Eudaimonic and hedonic orientations: Theoretical considerations and research findings. Handbook of Eudaimonic Well-Being.

[33] Diener E., Emmons R.A., Larsen R.J., Griffin S. (1985). The satisfaction with life scale. J. Personal. Assess..

[34] Watson D., Clark L.A., Tellegen A. (1988). Development and validation of brief measures of positive and negative affect: The PANAS scales. J. Personal. Soc. Psychol..

[35] Waterman A.S., Schwartz S.J., Zamboanga B.L., Ravert R.D., Williams M.K., Bede Agocha V., Yeong Kim S., Donnellan B. (2010). The Questionnaire for Eudaimonic Well-Being: Psychometric properties, demographic comparisons, and evidence of validity. J. Posit. Psychol..

[36] Preacher K.J., Hayes A.F. (2004). SPSS and SAS procedures for estimating indirect effects in simple mediation models. Behav. Res. Methods Instrum. Comput..

[37] Hayes A.F. (2017). Introduction to Mediation, Moderation, and Conditional Process Analysis: A Regression-Based Approach.

[38] Braaten A., Huta V., Tyrany L., Thompson A. (2019). Hedonic and eudaimonic motives toward university studies: How they relate to each other and to well-being derived from school. J. Posit. Sch. Psychol..

[39] Bujacz A., Vittersø J., Huta V., Kaczmarek L.D. (2014). Measuring hedonia and eudaimonia as motives for activities: Cross-national investigation through traditional and Bayesian structural equation modeling. Front. Psychol..

[40] Asano R., Igarashi T., Tsukamoto S. (2020). The Hedonic and Eudaimonic Motives for Activities: Measurement Invariance and Psychometric Properties in an Adult Japanese Sample. Front. Psychol..

[41] Ford B.Q., Dmitrieva J.O., Heller D., Chentsova-Dutton Y., Grossmann I., Tamir M., Uchida Y., Koopmann-Holm B., Floerke V.A., Uhrig M. (2015). Culture shapes whether the pursuit of happiness predicts higher or lower well-being. J. Exp. Psychol. Gen..

